# Data set on optimized biodiesel production and formulation of emulsified *Eucalyptus teriticornisis* biodiesel for usage in compression ignition engine

**DOI:** 10.1016/j.dib.2018.07.053

**Published:** 2018-07-26

**Authors:** V. Hariram, R. Prakash, S. Seralathan, T. Micha Premkumar

**Affiliations:** Department of Mechanical Engineering, Hindustan Institute of Technology and Science, Chennai, Tamilnadu, India

**Keywords:** Biodiesel, Transesterification, Emulsification, Taguchi׳s method, Catalyst concentration

## Abstract

This data article presents the experimental values pertaining to the bio-oil extraction, optimizing biodiesel production and formulation of emulsified fuel blends of *E.tereticornisis* bio-oil for its use in compression ignition engine. The *E.tereticornisis* leaves were collected from the interior region of Puducherry, India. Soxhlet extraction process, in the presence of n-hexane, yielded 5.2% of bio-oil. Based on the free fatty acid content, base catalysed transesterification process was adopted along with use of sodium hydroxide and methanol. Optimization of biodiesel yield was carried out by varying the operating parameters. A biodiesel yield of 74.19% was obtained at eighty minutes reaction duration, 1.8 l/g ms of sodium hydroxide, 70 °C reaction temperature and 8:1 oil to molar ratio. Furthermore, the physiochemical properties improved by emulsifying the obtained biodiesel with 5% of water in presence of surfactant through experiments carried out based on Taguchi׳s DOE method.

## Specifications Table

**Table:** 

Subject area	Alternate fuels
More specific subject area	Biofuels
Type of data	Figures and Tables
How data was acquired	Experimental investigations in the biodiesel laboratory
Data format	Raw as well as tabulated
Experimental factor	Optimization of biodiesel production and emulsified fuel formulation based on Taguchi׳s DOE
Experimental features	Bio-oil extraction through solvent extraction method using soxhlet apparatus and water based biodiesel emulsification.
Data sources	Antoine Lavoisier Fuels and Lubricants Laboratory, Hindustan Institute of Technology and Science
Data accessibility	Data is along with this article

## Value of the data

• This data set illustrates the methodology to extract bio-oil from *E.tereticornisis* through solvent extraction method.
• Biodiesel production is optimized by varying the reaction duration, reaction temperature, catalyst concentration and molar ratio.
• Gas Chromatography Mass Spectrometry (GC/MS) analysis discloses the various FAME׳s present in the *E.tereticornisis* biodiesel.
• Emulsified biodiesel blends based on Taguchi׳s DOE technique enhance the physiochemical properties of these biodiesel blends.
• The physiochemical properties of *E.tereticornisis* bio-oil, biodiesel and its emulsified form are compared with mineral diesel to understand its suitability in CI engine.

## 1 Data

Based on the earlier studies [1], [2], [3], it was observed that emulsified eucalyptus biodiesel production was not extensively analysed by investigators. In this data article, the ability of using an emulsified eucalyptus biodiesel in a compression ignition engine (CI) was analysed. This could lead to a significant increase in the engine performance along with reduced exhaust emissions.

**Fig. 1:**
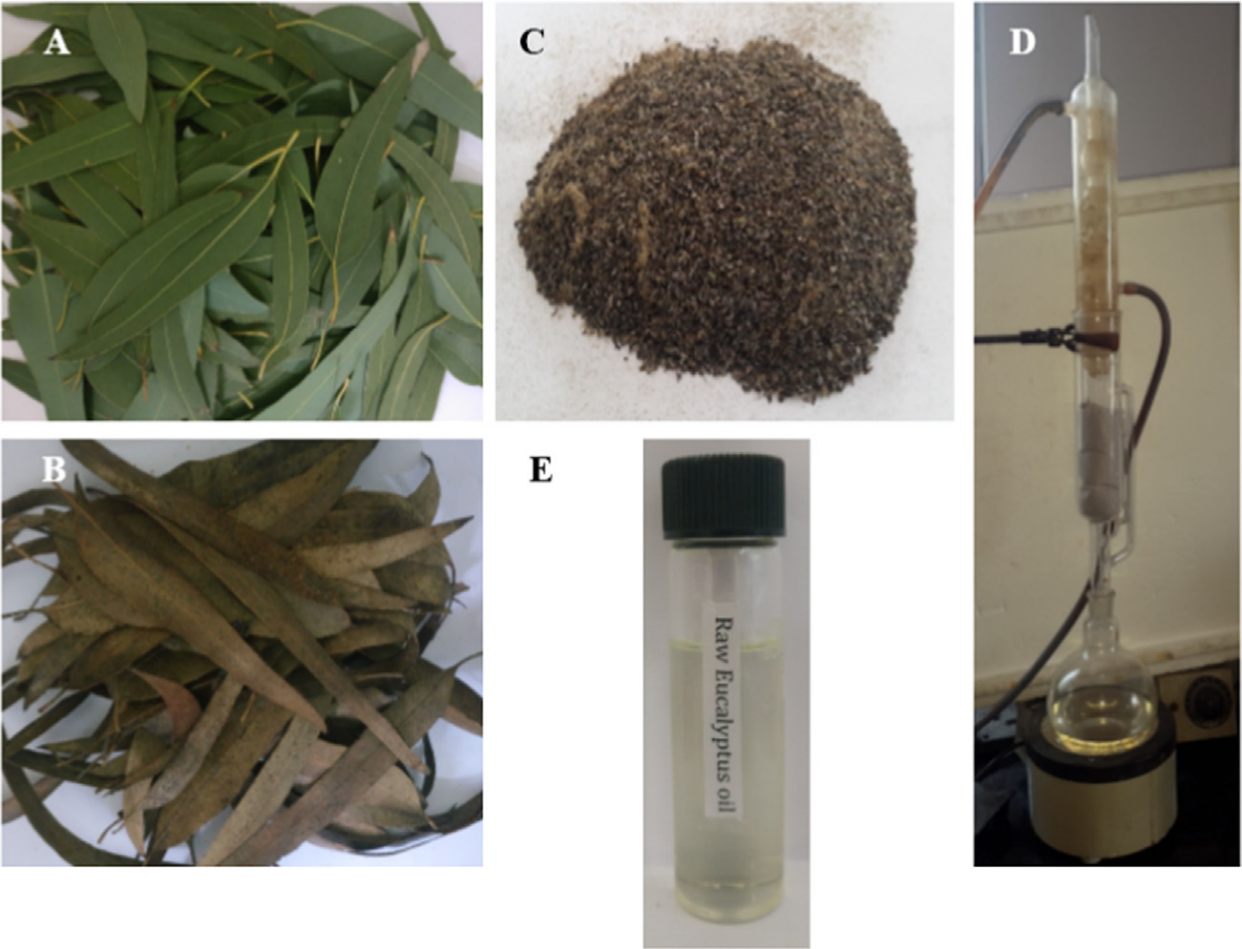
Extraction of bio-oil from *E.tereticornisis* through soxhlet extraction technique.

This data article reveals the methodology for extracting bio-oil from *Eucalyptus teriticornisis*, transforming the extracted bio-oil into biodiesel through transesterification, characterizing the biodiesel and emulsifying the biodiesel with water in presence of surfactant to enhance its physiochemical properties. Five types of data were presented in this data article. First, the bio-oil was extracted through solvent extraction method using soxhlet apparatus. The step-by-step pictorial representation was given in Fig. 1 in this data article. Second, the extracted bio-oil was subjected to base catalysed transesterification process to obtain *E.tereticornisis* biodiesel. A maximum yield of biodiesel was obtained by optimizing the operating parameters as given in Table 1. Third, the obtained biodiesel was characterized using GC/MS analysis to identify the various FAMEs present in the biodiesel. Fourth, the physiochemical properties of the biodiesel were enhanced by emulsifying it with water in presence of a surfactant. The stability analysis of this emulsified fuel blend was conducted using L_9_3^4^ orthogonal array of Taguchi׳s DOE approach as described in Tables 3 and 4. Finally, a comparative study of the physiochemical properties including *E.tereticornisis* bio-oil, biodiesel, and emulsified fuel blend with mineral diesel was also performed.

**Table 1:** Optimized biodiesel production from bio-oil of *Eucalyptus tereticornis*.

**Reaction duration (in minutes)**	**Catalyst concentration (grams/lts)**	**Reaction temperature (in °C)**	**Molar ratio**	**% yield of biodiesel**
60	1.2	50	6:1	42.16
70	1.5	50	7:1	51.70
80	1.8	50	8:1	52.23
90	2.1	50	9:1	52.79
60	1.2	60	6:1	43.59
70	1.5	60	7:1	52.43
80	1.8	60	8:1	53.70
90	2.1	60	9:1	58.36
60	1.2	70	6:1	48.79
70	1.5	70	7:1	55.76
**80**	**1.8**	**70**	**8:1**	**74.19**
90	2.1	70	9:1	73.12
60	1.2	80	6:1	51.62
70	1.5	80	7:1	57.31
80	1.8	80	8:1	57.83
90	2.1	80	9:1	70.18

## 2 Experimental design, materials and methods

### 2.1 Materials

Eucalyptus tereticornis trees grew abundantly in various parts of India. Extraction of eucalyptus bio-oil from its leaves could be carried out throughout the year. Therefore, its availability was perpetual and not a seasonal one. The bio-oil also acts as solubilizers which enhanced the ignition quality when blended with mineral diesel. Furthermore, it can be blended directly with diesel in esterified form without any modification in the existing CI engine.

*E.tereticornisis* leaves were collected from the interior region of Puducherry, India. Industrial grade n-hexane with 99% purity and methanol were procured from National Petro Chem, Chennai, Tamil Nadu, India. Astraa Chemical, Mumbai, India supplied the laboratory grade sodium hydroxide. Ultrasonicator bath was used to emulsify the biodiesel with water. Erlenmeyer flask, round or flat bottomed flask and side arm flask were the other apparatus used for oil extraction and transesterification [4].

### 2.2 Methods

In this bio-oil extraction process, the matured leaves produced 1.18 ml of bio-oil per 20 g with an oil extraction efficiency of 5.9%. On the other hand, the young tender leaves produced only 1.05 ml of bio-oil per 20 g. Therefore, matured leaves were found favourable for bio-oil extraction which an improved yield of 11.02% compared to tender *E.tereticornisis* leaves.

The collected matured leaves of *E.tereticornisis* was thoroughly cleaned in flowing water for the removal of adhered sand particles, if any and followed by cleansing with distilled water. The leaves were then sun dried for 72 h in ambient environment. Further, the removal of moisture was carried out using hot oven drying kept at 50–55 °C for 24 h. The absence of moisture content was ensured by weighing the raw material before and after the drying process. Mortar and pestle grinder was used to process the dried leaves as shown in Fig. 1C till the size of 1.5 mm was reached. Solvent extraction method using soxhlet apparatus was deployed for the extraction of bio-oil [5].

### 2.3 Soxhlet extraction process

400 ml of n-hexane solvent was placed in the round bottomed flask of the soxhlet apparatus. 20 g of processed *E.tereticornisis* was filled in the thimble and placed in the middle part of soxhlet apparatus. The apparatus was fitted with a condensation arrangement at the top as shown in Fig. 1D. The *n*-hexane solvent was heated upto 74 °C during which it vaporised and reached the top layer. Due to the condensation process, *n*-hexane was liquefied and it dropped down into the thimble at an operating temperature between 50 and 55 °C. The n-hexane solvent reacted with the processed *E.tereticornisis* rupturing the cell membrane thereby, expelling the lipid content from the biomass. *n*-hexane along with lipids (bio-oil) flowed downwards and occupied the bottom layer of the round bottomed flask forming a colloidal solution. On repeating this cycle up to 12–14 times, bio-oil as shown in the Fig. 1D, was collected at the bottom [6]. The maximum extraction of *E.tereticornisis* bio-oil was noticed to be 1.18 ml per 20 g of processed biomass at an extraction efficiency of 5.9%.

### 2.4 Single stage transesterification

The transesterification process reduced the kinematic viscosity of eucalyptus biodiesel but also infused the methyl esters to the fuel thereby enhancing the oxidization during combustion phenomenon. This helped in maximizing the conversion of fuel׳s energy into useful work. Also, reducing the kinematic viscosity up to 2.74 mm^2^/s reduced the fuel injector clogging effect. The transesterification process also improved the calorific value and cetane number upto 40.46 MJ/kg and 53.5 respectively thereby promoting better combustion. Also, the FFA content was reduced up to 0.95% favouring its use as a CI engine fuel.

**Fig. 2:**
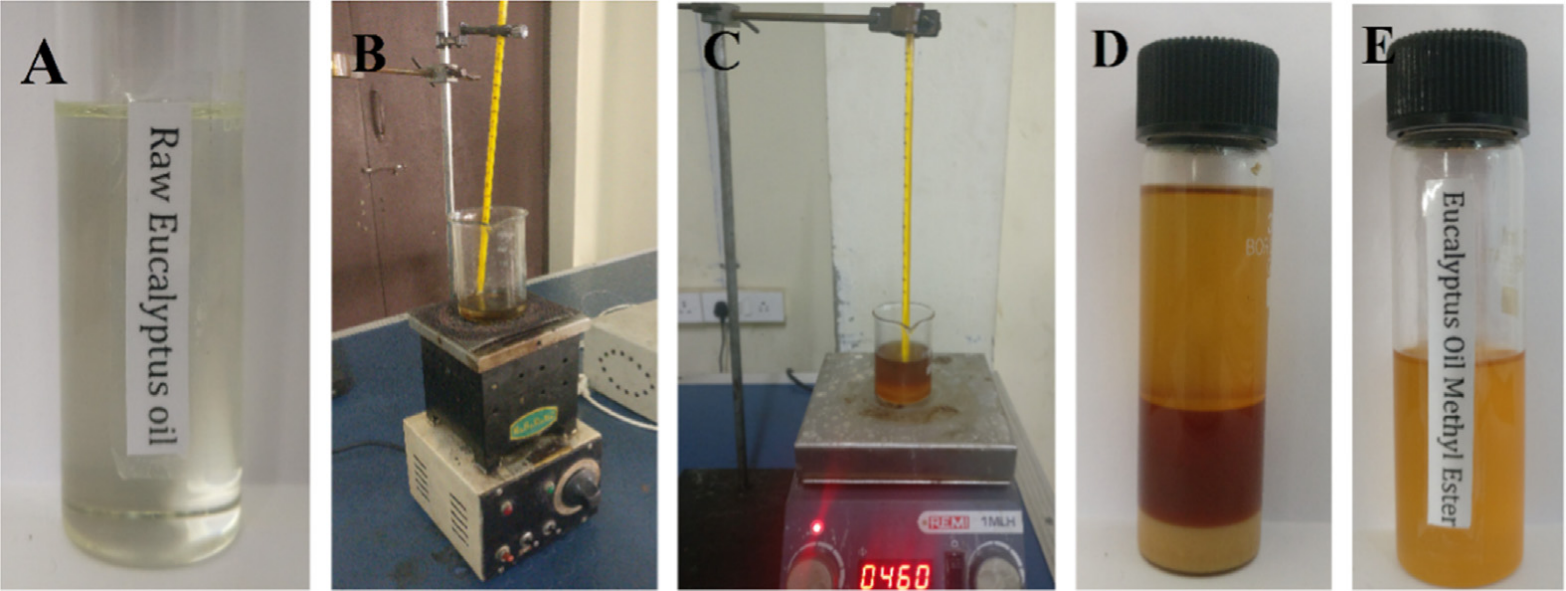
Single stage transesterification process to derive *E.tereticornisis* biodiesel.

Titration based analytical approach showed the acid value of *E.tereticornisis* bio-oil as 1.72%. Therefore, base catalysed transesterification was chosen as it was one of the best methods for reducing the viscosity using sodium hydroxide and methanol. Experimental trials were conducted by varying the operating variables like reaction duration (60–90 min), reaction temperature (50–80 °C), catalyst concentration (1.2–2.1 g/lts) and molar ratio (6:1–9:1). Fig. 2 shows the typical steps in single stage transesterification process to derive *E.tereticornisis* biodiesel. Table 1 indicates that the maximum yield of biodiesel (i.e., 74.19%) was at 80 minutes reaction duration with 1.8 g/lts catalyst concentration, 70 °C reaction temperature and 8:1 M ratio [7].

### 2.5 Gas chromatography mass spectrometry analysis

**Fig. 3:**
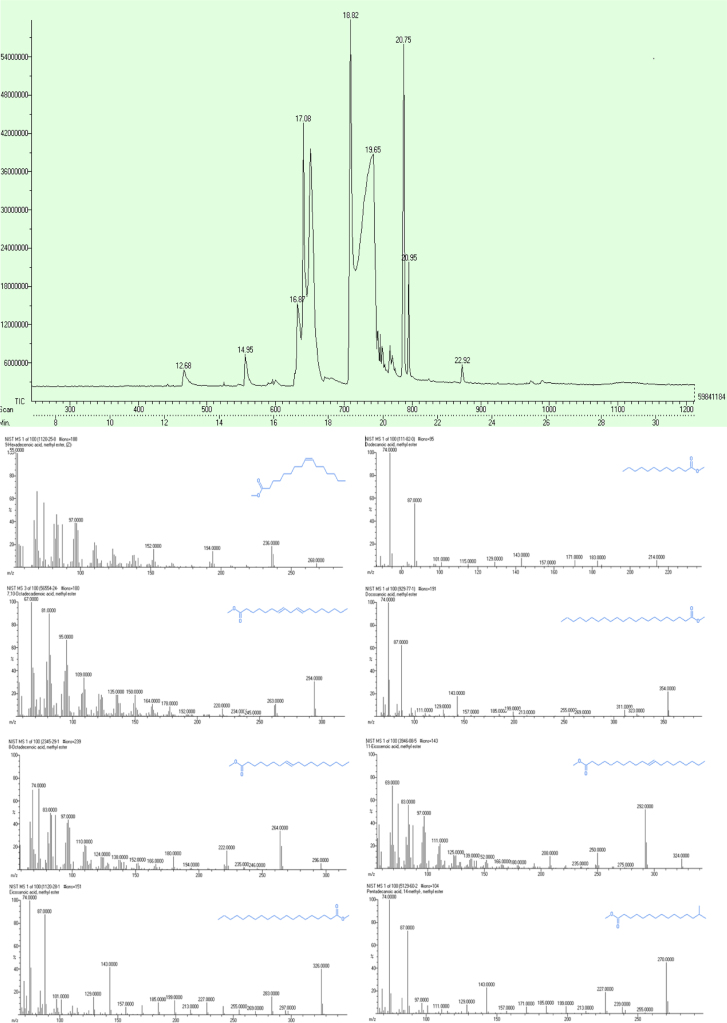
GC/MS chromatogram and fragmentation patterns of *E.tereticornisis* biodiesel *Emulsified fuel formulation*.

JOEL Gate GC Mate II data system was used to identify the various FAME׳s present in the *E.tereticornisis* biodiesel. NIST MS2 library confirmed the presence of Lauric acid at RT12.68, Myristic acid at RT14.95, Palmitoleic acid at RT16.87, Pentadecyclic acid at RT17.18, Linoleic acid at RT18.82, Oleic acid at RT19.65, Arachidic acid at RT20.98, Gondoic acid at RT20.78 and Behenic acid at RT22.92. Table 2 and Fig. 3 depicts the results of GC/MS analysis and the presence of various FAME׳s based on their fragmentation patterns [8].

**Table 2:** Fatty acid methyl esters in Eucalyptus biodiesel.

**Peak no**	**Retention time**	**Name of the ester**	**Name of the fatty acid**
1	12.68	Dodecanoic acid, methyl ester	Lauric acid
2	14.95	Methyl tetradecanoate	Myristic acid
3	16.87	9-Hexadecenoic acid, methyl ester,	Palmitoleic acid
4	17.18	Pentadecanoic acid, 14-methyl-methyl ester	Pentadecyclic acid
5	18.82	7,10 Octadecadienoic acid, methyl ester	Linoleic acid
6	19.65	8-Octadecenoic acid, methyl ester	Oleic acid
7	20.98	Eisosanoic acid, methyl ester	Arachidic acid
8	20.78	11.Eicosenoic acid, methyl ester	Gondoic acid
9	22.92	Docosanoic acid, methyl ester	Behenic acid

### 2.6 Formulation of emulsified fuel

Formulation of emulsified fuel was done using *E.tereticornisis* biodiesel-mineral diesel blend with water in presence of a surfactant (Span 80). The various concentration levels of the emulsified fuel formulations are given in Table 3. Ultrasonication performed in a sonicator bath at 4000 rpm accomplished through mixing and emulsion formation. Lower hydrophilic-lipophilic balance value was also taken into consideration to make this water in oil emulsion. L^9^3_4_ orthogonal array of Taguchi׳s design of experiments approach was implemented to formulate the emulsified fuel blend which reduced the real time combinations of experiments to be performed from 81 to nine as shown in Tables 3 and 4. The fuel stability inspection was conducted by observing the emulsified fuel blends on daily basis. The experiment no. 8 with concentration levels (volume based) of mineral diesel, eucalyptus biodiesel, water and surfactant at 85, 20, 5 and 6 respectively was found to be stable (i.e., highly miscible) up to 21 days [9].

**Table 3:** Taguchi table for L_9_3^4^fuel formulation.

**Variable parameters**	**Levels of concentration**		
	**L1**	**L2**	**L3**
Mineral diesel	75	80	85
Eucalyptus biodiesel	15	20	25
Water	5	10	15
Surfactant	2	4	6

**Table 4:** L9 orthogonal array for emulsified eucalyptus biodiesel test fuel.

**Experiment no**	**Concentration levels and its parameters**				
	**Mineral diesel**	**Eucalyptus biodiesel**	**Water**	**Surfactant**	**Fuel stability (in days)**
1	75	15	5	2	4
2	75	20	10	4	1
3	75	25	15	6	2
4	80	15	10	6	2
5	80	20	15	2	6
6	80	25	5	4	4
7	85	15	15	4	5
**8**	**85**	**20**	**5**	**6**	**21**
9	85	25	10	2	3

Afterwards, a minor sedimentation layer was noticed at the bottom of the mixture container. This could be overcome by performing ultrasonication of the mixture again at 4000 rpm for 60 min. It is to mention that the nine experiments based on Taguchi׳s design of experiments approach were repeated three times in the laboratory conditions to ensure the repeatability of the values and the averaged values were taken into consideration in this present study.

### 2.7 Physiochemical properties

Calorific value, also termed as heating value, is the measure of heat produced by burning a specified quantity of hydrocarbon. ASTM D5865 method was used to determine the calorific value of *E.tereticornisis* biodiesel. RSB3 HAMCA bomb calorimeter was used to identify the gross calorific value. 25 ml of *E.tereticornisis* biodiesel is placed in a closed environment inside a crucible of bomb calorimeter which was kept under pressurized condition (30–35 atm.). Excess oxygen was supplied along with electric current which initiated the ignition of *E.tereticornisis* biodiesel inside the bomb calorimeter. The variation in combustion temperature was used to determine the calorific value and the value was estimated as 40.46 MJ/kg and 41.24 MJ/kg for *E.tereticornisis* biodiesel and emulsified *E.tereticornisis* biodiesel blend [1]. Kinematic viscosity is a parameter which illustrates the ability of the fluid to flow. ASTM D445 method was used to measure the kinematic viscosity of *E.tereticornisis* using IP70 redwood viscometer. The oil cup of the viscometer was filled with 40 ml of *E.tereticornisis* biodiesel and it is thermally equipoised at 40 °C by surrounding it in a water bath along with constant stirring action. The kinematic viscosity was estimated by allowing each test fuel to travel separately inside the kohlrausch flask and the values were found to be 3.92 mm^2^/s, 2.74 mm^2^/s and 2.69 mm^2^/s for *E.tereticornisis* bio-oil, biodiesel and its emulsified fuel blend respectively.

ASTM D3278 method was adopted to understand the flammability limits of *E.tereticornisis* bio-oil, its biodiesel and emulsified fuel blend. P20 Abel flash point apparatus was used to determine the flash point. The oil cup was filled with 50 ml of fuel and the temperature was raised up to 80 °C using a heating element in a thermally stabilized environment with constant mechanical stirring at a rate of 70 rpm. The time duration for every 1 °C rise in temperature was recorded to analyse the flash point. Based on the experiments, it was found that the flash point for *E.tereticornisis* bio-oil and its biodiesel as 112 °C and 102 °C respectively. Mettler TOLEDO Densometer was used to determine the density of test fuels using ASTM D792 method. The density of *E.tereticornisis* bio-oil and *E.tereticornisis* biodiesel was estimated as 922 kg/m^3^ and 905 kg/m^3^ respectively.The ignition quality of *E.tereticornisis* biodiesel was found by calculating the cetane indices using ASTM D613 method. The cetane number of *E.tereticornisis* biodiesel and its emulsified fuel blend was found to be 53.50 and 52 respectively. Table 5 lists the various physiochemical properties of all the test fuels [4].

**Table 5:** Comparison of physiochemical properties – *E. tereticornis* bio-oil and its biodiesel.

**Properties**	***E. tereticornis*****bio-oil**	***E. tereticornis*****biodiesel**	**Emulsified*****E. tereticornis*****biodiesel-diesel blend**	**ASTM standards**
Density (kg/m^3^)	922	905	891	ASTM D792
Specific gravity @ 25 °C (g/cm^3^)	0.945	0.891	0.882	ASTM D1963
Kinematic viscosity @ 40 °C (mm^2^/s)	3.92	2.74	2.69	ASTM D445
Cetane number	44.5	53.5	52	ASTM D613
Calorific value (MJ/kg)	36.15	40.46	41.24	ASTM D5865
Flash point (°C)	112	102	104	ASTM D3278
Acid value(mg KoH/gm)	2.02	0.24	0.22	ASTM D1980
FFA content (%)	1.52	0.95	0.93	ASTM D6751
