# Supplementary material for: Data set on optimized biodiesel production and formulation of emulsified Eucalyptus teriticornisis biodiesel for usage in compression ignition engine
Source: Data Brief. 2018 Jul 26;20:6–13. doi: 10.1016/j.dib.2018.07.053 (PMC6083299; doi:10.1016/j.dib.2018.07.053)
Supplement: Supplementary file 1 — Supplementary material [file mmc1.docx]

**Conflict of Interest form**

**Data set on Optimized Biodiesel Production and Formulation of Emulsified Eucalyptus teriticornisis Biodiesel for usage in Compression Ignition Engine**

Hariram V*, Prakash S, Seralathan S, Micha Premkumar T

Department of Mechanical Engineering

Hindustan Institute of Technology and Science, Chennai, Tamilnadu, India

* Corresponding author: connect2hariram@gmail.comManuscript No: DIB-D-18-01458

***The authors would like to state that there is no potential conflict of interest with the work presented in this data article.***
